# Postmortem chest computed tomography in COVID-19: A minimally invasive autopsy method

**DOI:** 10.1016/j.ejro.2024.100546

**Published:** 2024-01-13

**Authors:** Paulo Savoia, Marcio Valente Yamada Sawamura, Renata Aparecida de Almeida Monteiro, Amaro Nunes Duarte-Neto, Maria da Graça Morais Martin, Marisa Dolhnikoff, Thais Mauad, Paulo Hilário Nascimento Saldiva, Claudia da Costa Leite, Luiz Fernando Ferraz da Silva, Ellison Fernando Cardoso

**Affiliations:** a Department of Radiology, University of Sao Paulo School of Medicine, Institute of Radiology, Rua Doutor Ovidio Pires de Campos, 75, 05403-010, Cerqueira Cesar, São Paulo, SP, Brazil; b Department of Pathology, University of Sao Paulo School of Medicine, Av. Dr. Arnaldo, 455, sala 1155, 01246-903, Cerqueira Cesar, São Paulo, SP, Brazil; c Hospital Israelita Albert Einstein, Av. Albert Einstein, 627/701, 05652-900, Morumbi, São Paulo, SP, Brazil

**Keywords:** Tomography, Thorax, Autopsy, Pathology

## Abstract

**Objectives:**

Performing autopsies in a pandemic scenario is challenging, as the need to understand pathophysiology must be balanced with the contamination risk. A minimally invasive autopsy might be a solution. We present a model that combines radiology and pathology to evaluate postmortem CT lung findings and their correlation with histopathology.

**Methods:**

Twenty-nine patients with fatal COVID-19 underwent postmortem chest CT, and multiple lung tissue samples were collected. The chest CT scans were analyzed and quantified according to lung involvement in five categories: normal, ground-glass opacities, crazy-paving, small consolidations, and large or lobar consolidations. The lung tissue samples were examined and quantified in three categories: normal lung, exudative diffuse alveolar damage (DAD), and fibroproliferative DAD. A linear index was used to estimate the global severity of involvement by CT and histopathological analysis.

**Results:**

There was a positive correlation between patient mean CT and histopathological severity score indexes - Pearson correlation coefficient (R) = 0.66 (p = 0.0078). When analyzing the mean lung involvement percentage of each finding, positive correlations were found between the normal lung percentage between postmortem CT and histopathology (R=0.65, p = 0.0082), as well as between ground-glass opacities in postmortem CT and normal lungs in histopathology (R=0.65, p = 0.0086), but negative correlations were observed between ground-glass opacities extension and exudative diffuse alveolar damage in histological slides (R=−0.68, p = 0.005). Additionally, it was found is a trend toward a decrease in the percentage of normal lung tissue on the histological slides as the percentage of consolidations in postmortem CT scans increased (R =−0.51, p = 0.055). The analysis of the other correlations between the percentage of each finding did not show any significant correlation or correlation trends (p ≥ 0.10).

**Conclusions:**

A minimally invasive autopsy is valid. As the severity of involvement is increased in CT, more advanced disease is seen on histopathology. However, we cannot state that one specific radiological category represents a specific pathological correspondent. Ground-glass opacities, in the postmortem stage, must be interpreted with caution, as expiratory lungs may overestimate disease.

## Highlights

• Radiologists can read COVID-19 postmortem chest CT aiding pathologists in forensic studies.
• Normal postmortem chest CT scans correlates to normal lung histopathology (screening tool?).
• COVID-19 postmortem imaging findings correlate in extension and severity to histopathology.

## 1 Introduction

The number of fatal victims of COVID-19 pandemic has reached approximately 6.9 million worldwide (up to March 2023) [1]. Despite the elevated number of deaths, literature regarding COVID-19 conventional autopsies was relatively scarce, mostly because of contagion risks and/or the strict protection procedures recommended, which has substantially reduced traditional necropsies [2], [3]. In our country, conventional autopsies were forbidden when the pandemic broke out [4]. This scenario will be observed in the beginning of any pandemic and the need for alternative methods is crucial. Autopsy operational issues drove some authors to develop minimally invasive autopsy methods to study fatal COVID-19: some performed lung, heart, liver and spleen histopathological analysis by using blinded tissue sampling [5], and others used lung ultrasound to guide histopathological analysis [6], [7].

Chest CT was proven to be the method of choice to assess COVID-19 lung involvement in living patients, endorsed by the Society of Thoracic Radiology, the American College of Radiology, and the Radiological Society of North America [8]. However, the literature about postmortem CT in COVID-19 is limited. Some authors have used postmortem CT as a triage tool to refer a patient to conventional autopsy when there were no typical CT findings of COVID-19 lung involvement to minimize the risks of autopsy team contagion [9], [10]. A case series analysis has shown that despite postmortem chest CT limitations (mainly expired lungs and hypostasis), most classic COVID-19 findings, as well as eventual additional findings, are present and similar to premortem chest CT. In some cases, authors could even demonstrate disease progression [11].

Our motivation emerged from the fact that autopsies were restricted and occasionally prohibited in the beginning of the COVID-19 pandemic, limiting the study of a recent novel disease. The development of an alternative method to traditional autopsies, such as a minimally invasive autopsy seemed to be a solution to satisfy the eagerness to comprehend the pathophysiological processes involved in fatal COVID-19 cases.

Our main goal was to evaluate the additional value of postmortem chest CT to minimally invasive autopsy and verify whether postmortem CT lung findings could provide information regarding histopathological involvement.

## 2 Materials and methods

This study was approved by our National Research Ethical Committee (CONEP CAAE 30364720.0.0000.0068).

### 2.1 Population

From April to June 2020 (during the ‘first wave’ of the COVID-19 pandemic), 29 patients died with a laboratory confirmation of COVID-19 and had an autopsy requested by our institution’s medical staff after informed consent was obtained from the next of kin.

### 2.2 Minimally invasive autopsy protocol

The deceased bodies were covered by a plastic safety bag and transported to the morgue by nursery staff with adequate personal protective equipment. No inflation of the lungs was performed. The autopsy service performs postmortem studies with a dedicated CT scanner. Two trained technicians prepared the body for the CT scan, wrapping it with an additional plastic bag. The CT operator never had direct contact with the bodies; in fact, most times, the equipment was operated remotely from another central operation room in the hospital.

Postmortem chest CTs were performed using a Somatom Emotion scanner (Siemens Healthineers, Erlangen Germany). Images were saved in DICOM™ format, with a standard lung filter reconstruction applied. CT Scan parameters are displayed on Table 1. All postmortem studies were sent to an online Picture Archiving and Communication System (PACS), provided by Purview©. All CTs were performed less than 24 h after death.

**Table 1:** Postmortem Chest CT Scan Parameters.

Parameter	Value
kV	130
mA	AEC (78 to 260) or fixed in 128 or 136
Pitch	0.8
Matrix	512 × 512
Detector Rows	16
Detector Thickness	1.2 mm
Collimation	19.2 mm
Slice Thickness	1.5 mm

(AEC = automatic exposure control).

**Fig. 1:**
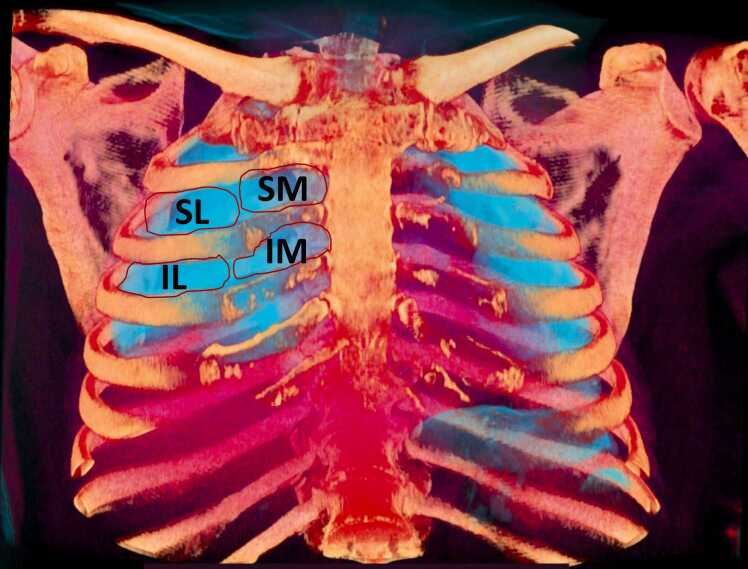
3D volume rendering reformat from a postmortem chest CT from our study (using RadiAnt™ DICOM Viewer 2022.1 Software – 3D Preset: Bones and Skin 3) shows how the postmortem expired lungs (in blue) have their anterior lower limits around the 4th or 5th intercostal spaces. From this reconstruction, it is possible to understand why the 2nd anterior intercostal space was used for the tissue sample collection of the upper regions and the 3rd anterior intercostal space was used for the tissue sample collection of the inferior regions. The limit between the lateral and medial regions was the midclavicular line. The 4 regions of access to tissue sample collection are also shown: SL = superolateral; SM = superomedial; IL = inferolateral; IM = inferomedial.

Immediately after the postmortem chest CT was performed, multiple tissue samples were collected by ultrasound-guided biopsies, including samples from the brain, lungs, heart, liver, spleen, kidneys, salivary glands, pancreas, testis and striated muscle. This work considers only lung samples. They were ultrasound guided only to certify that lung tissue was sampled. Furthermore, the biopsies were obtained in a standardized protocol from 4 different regions in each lung (Fig. 1): superolateral, superomedial, inferolateral and inferomedial. As postmortem lungs are usually expired, anteriorly, we found the inferior lung limits around the 4th or 5th intercostal space; therefore, the 3rd sternum costal joint was used as the division mark between the superior and inferior regions. Regarding lateral and medial regions, the division marks were the midclavicular lines. The posterior and anterior areas of each region were tissue sampled. We used a portable SonoSite M-Turbo R Ultrasound (Fujifilm, Bothell, WA, USA) with broadband and multifrequency transducers, C60x (5–2 MHz Curved) and HFL38X (13–6 MHz Linear), and semiautomatic coaxial 14 G needles, 20 centimeters in length. The needles reached the posterior chest wall to certify that the posterior regions were always included. Approximately 60 pulmonary samples were collected from each lung.

### 2.3 Chest CT analysis

**Fig. 2:**
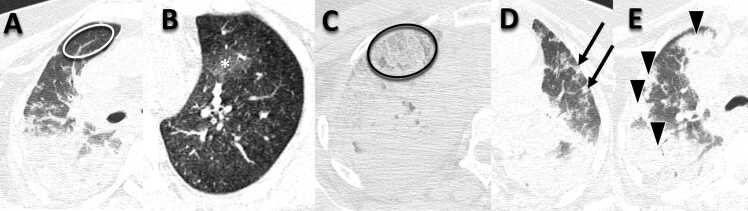
Examples of postmortem CT findings of our sample: A) Normal lung parenchyma (white ellipse). B) Ground-glass opacities (white asterisk). C) Crazy paving (black ellipse). D) Small consolidations (black arrows). E) Large consolidations (black arrowheads).

Images were examined independently by two experienced thoracic radiologists (each with more than 10 years of experience in specialized practice) who were blinded to the histopathological findings, and disagreements were resolved by consensus. Each region corresponding to the biopsied regions described above was quantified and classified into the following categories: normal; ground-glass opacities; crazy-paving; small consolidations; and large or lobar consolidations, as shown in Fig. 2.

The following radiological criteria were used to define each of the five categories above, based on the Fleischner Society glossary of terms for thoracic imaging [12]: (a) normal: preserved parenchyma; (b) ground-glass opacities: hazy increased opacity of lung, with preservation of bronchial and vascular margins; (c) crazy-paving: thickened interlobular septa and intralobular lines superimposed on a background of ground-glass opacity, resembling irregularly shaped paving stones; (d) small consolidations: a homogeneous increase in pulmonary parenchymal attenuation that obscures the margins of vessels and airway walls (up to 1.0 cm); (e) large or lobar consolidations: a homogeneous increase in pulmonary parenchymal attenuation that obscures the margins of vessels and airway walls (larger than 1.0 cm). An air bronchogram may be present.

The radiologists subjectively quantified the proportion of the five different patterns in each region (expressed as 5 % ranges – up to 20; 40; 60; 80; and 100 %). The most severe diagnosis of each region was also highlighted. A consensus of the analysis was reached.

Other quantitative and qualitative fatal COVID-19 postmortem chest CT findings were also analyzed: percentage of global lung involvement; uni/bilaterality; affected lobes; posterior or inferior predominance; peripheral, central or mixed distribution; presence of emphysema, fibrosis, mediastinal lymph node enlargement, pleural effusion, pleural thickening, pneumothorax, pericardial effusion, pneumomediastinum, aortic calcifications, coronary calcifications, and subcutaneous emphysema.

### 2.4 Histological analysis

**Fig. 3:**
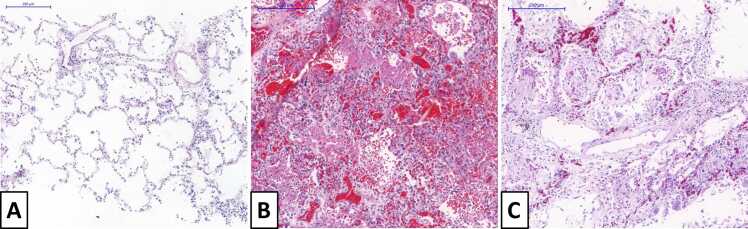
Examples of postmortem histological findings of our sample: A) Normal lung parenchyma. B) Acute/Exudative Diffuse Alveolar Damage. C) Fibroproliferative diffuse alveolar damage.

Slides were examined with consensus by five experienced pulmonary pathologists (with a minimum of 6 and a maximum of 30 years of specialized practice experience) blinded to radiological findings and classified into the following categories: normal lung, exudative diffuse alveolar damage (DAD) and fibroproliferative DAD, as illustrated in Fig. 3.

The following histological criteria were used to define each pattern [13]: (a) normal lung: preserved architecture, without inflammation, edema or exudate; (b) exudative DAD: interstitial and/or intraalveolar edema, interstitial inflammation, variable amounts of alveolar hemorrhage and fibrin deposition, intra-alveolar hyaline membranes and type II pneumocyte hyperplasia. Foci of neutrophilic pneumonia were also included in the acute/exudative pattern; and (c) fibroproliferative DAD: any degree of fibroblastic proliferation within the *interstitium* and/or alveolar spaces, including loose aggregates of fibroblasts admixed with scattered inflammatory cells, collagen deposition, squamous metaplasia, and possible remnants of hyaline membranes. The most severe diagnosis of each region was also highlighted, as it was not always the most common.

If the histopathological analysis exhibited any indications of secondary pneumonia, exclusion of the patient was necessary. This precaution ensured the ability to unequivocally attribute the findings solely to COVID-19, mitigating potential confounding factors.

The pathologists subjectively quantified the proportion of the three different patterns in each region corresponding to the biopsied regions described above (expressed as 5 % ranges – up to 20; 40; 60; 80; and 100 %). The most severe diagnosis of each region was also highlighted. A consensus of the analysis was reached.

Conventional autopsy of the lungs was not performed because it was forbidden in our country during the development of this study [4].

### 2.5 Statistical analysis

Statistical analysis was performed using R software (http://www.R-project.org). For each region, we calculated the Kappa concordance coefficient for the most severe diagnosis and the categorical percentage estimation between CT and the corresponding histological analysis, as follows: normal CT to normal pathology; ground-glass opacities to exudative DAD; crazy-paving, small consolidations, and large or lobar consolidations to fibroproliferative DAD. In addition, for each region, we calculated the Pearson correlation coefficient between each CT finding and histopathological finding.

To quantify lung pathological involvement in COVID-19, we proposed CT and histopathological severity scores as a linear combination of the percentage of pulmonary involvement weighted by the severity of the change. In the CT severity score (CTSS), the weighted coefficients for larger/confluent consolidations, small consolidations, crazy paving, and ground-glass opacities were 4, 3, 2, and 1, respectively. In the histological severity score (HSS), the weighted coefficients for fibroproliferative DAD and exudative DAD were 4 and 2, respectively, as follows:

$$\mathrm{CTSS}=4\left(\%\mathrm{big}\;\mathrm{or}\;\mathrm{confluence}\;\mathrm{consolidations}\right)+3\left(\%\mathrm{small}\;\mathrm{consolidations}\right)+2\left(\%\mathrm{crazy}\;\mathrm{paving}\right)+1(\%\mathrm{extensive}\;\mathrm{ground}\;\mathrm{glass}\;\mathrm{opacities})$$

$$\mathrm{HSS}=4\left(\%\mathrm{fibroproliferative}\;\mathrm{DAD}\right)+2(\%\mathrm{exudative}\;\mathrm{DAD})$$

These scores increase as the severity and extent of pulmonary involvement by COVID-19 increase, ranging from zero to 400. In addition, it enables the calculation of the correlation coefficient between scores. The scores were calculated for each of the 4 regions of each lung, and the mean score of each patient was taken for correlation.

## 3 Results

**Fig. 4:**
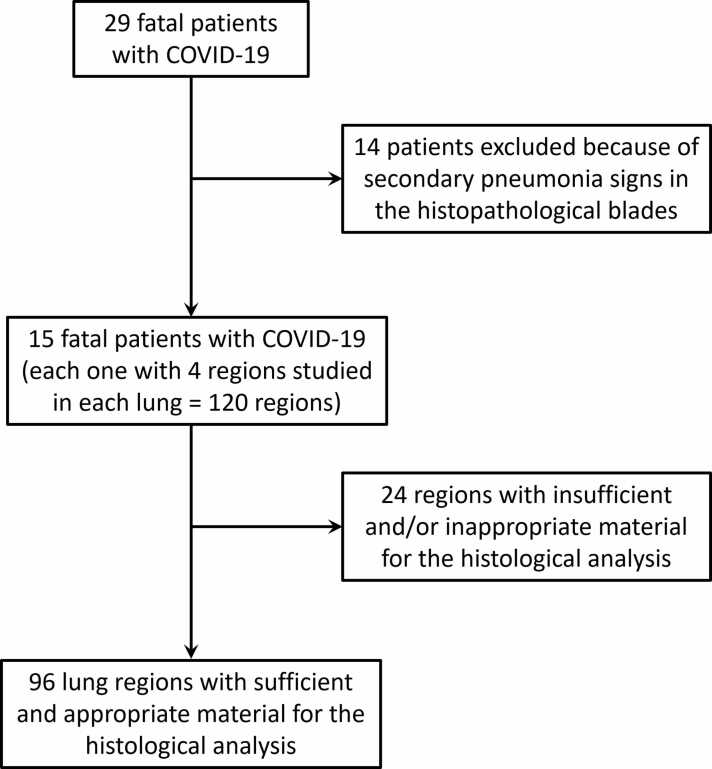
Flow diagram showing the initial number of patients and patients excluded. Afterward, from the patients not excluded, some regions had to be excluded because of insufficient and/or inappropriate material. In the end, 96 lung regions were analyzed.

Of the 29 patients who underwent the minimally invasive autopsy protocol described, 14 had signs of secondary pneumonia in the histological analysis and thus had to be excluded once CT findings could be related to an agent other than SARS-CoV-2. Table 2 shows the epidemiological and clinical data of the 29 studied patients. Of the 15 patients without signs of secondary pneumonia in the histopathological examination, not all of them had sufficient and appropriate material from the 4 regions of each lung for the histological analysis. Therefore, in the end, 96 regions of adequate tissue were sampled from these 15 patients (Fig. 4). The consensus agreement between the two radiologists about the chest CT analysis was 100 % for the most severe diagnosis of each region and 97.66 % for the proportion of the five different patterns in each region. The 2.33 % disagreement was totally resolved by reanalyzing it together and reaching consensus. The time between death and postmortem CT was 14h36m ± 06h38m (average ± standard deviation).

**Table 2:** Clinical and Demographic Characteristics of our cohort that was composed of 29 patients with fatal COVID-19. Data are displayed as the mean ± standard deviation. ICU: Intensive Care Unit.

Demographics and Clinical Characteristics of patients with fatal COVID-19		
	**No Secondary Pneumonia**	**Secondary Pneumonia (excluded)**
Patients (number)	15	14
Gender (%)	40 Male/60 Female	57.1 Male/42.9 Female
Age (years)	51.27 ± 16.05	56.79 ± 17.85
Weight (kg)	75.79 ± 15.50	73.10 ± 29.34
Height (m)	1.70 ± 0,07	1.69 ± 0.09
BMI (kg/m²)	25.88 ± 4.15	25.34 ± 9.02
Ethnicity (%)	86.7 White/6.7 Black/6.7 Brown	85.7 White/7.1 Black/7.1 Brown
Time from Symptoms Onset to Admission (days)	9.13 ± 6.62	6.29 ± 3.79
Time from Symptoms Onset to Death (days)	25.13 ± 9.96	20.29 ± 10.97
Time from Admission to Death (days)	16.0 ± 10.51	14.0 ± 10.10
Time from ICU Admission to Death (days)	11.07 ± 9.07	10.93 ± 9.23

There was no concordance between the CT and histopathological estimates of extension in all categories evaluated or between the most severe diagnosis for each region in CT and in the histological slides.

**Fig. 5:**
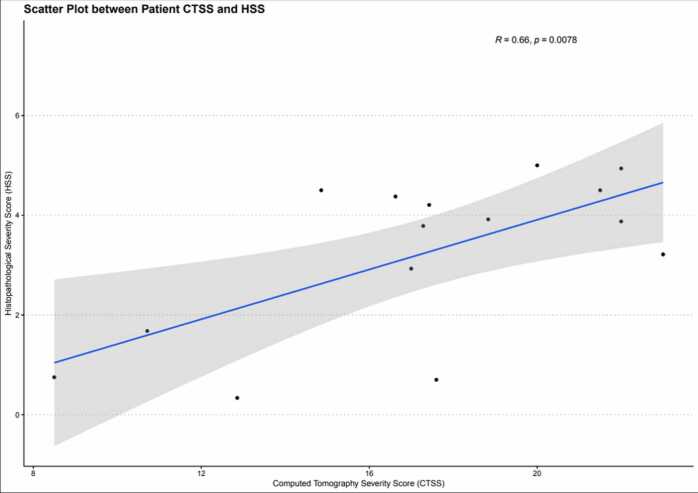
**:** The correlation between CT and Histopathological severity score indexes.

There was a positive correlation between the patient mean CT and histopathological severity score indexes. The Pearson correlation coefficient ®, estimated in our sample, was 0.66 (p = 0.0078), as shown in Fig. 5 (Scatter plot).

**Fig. 6:**
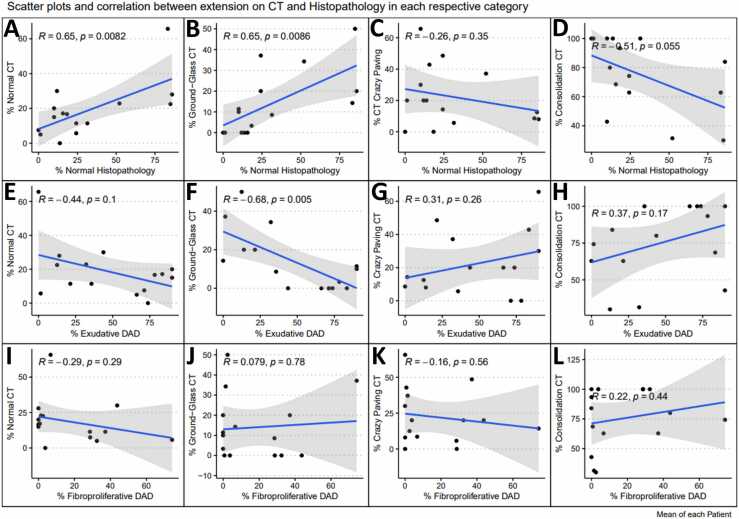
**:** Correlation - Mean lung involvement percentage in the blades and in the CT for each finding.

Furthermore, we calculated the mean lung involvement percentage in the slides and in the CT for each finding (grouping small and large consolidations in postmortem CT) in each patient and estimated the Pearson correlation coefficient (R) between lung involvement percentages, as shown in a set of graphs (Fig. 6). In Fig. 6, there is a positive correlation between the percentage of normal lung in postmortem CT and histological slides (R = 0.65, p = 0.0082) (6 A), as well as between the percentage of ground-glass opacities in postmortem CT scans and histological slides (R = 0.65, p = 0.0086) (6B). Moreover, as the percentage of ground-glass opacities in postmortem CT increased, there was a decrease in the percentage of exudative diffuse alveolar damage on the histological slides (negative correlation – R = −0.68, p = 0.005) (6 F). Additionally, in Fig. 6, there is a trend toward a decrease in the percentage of normal lung tissue on the histological slides as the percentage of consolidations in postmortem CT scans increases (negative correlation – R = −0.51, p = 0.055) (6D). The other subgraphs did not show any significant correlation or correlation trends (p ≥ 0.10).

**Fig. 7:**
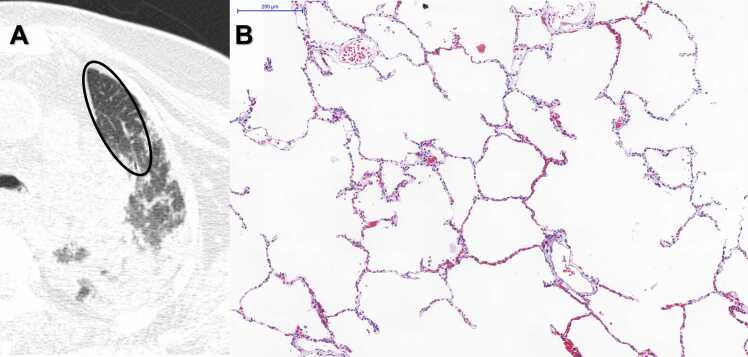
A) Postmortem Chest CT Image, Axial View, Left Upper Regions. There is a large area of normal lung parenchyma (ellipse). B) Histopathological sample of the same regions: Photomicrograph showing normal lung with open airspaces, thin alveolar septa, no inflammation and mild capillary congestion, commonly seen in autopsy specimens - HE Staining. Scale Bar - 200 µm - Objective 10x.

**Fig. 8:**
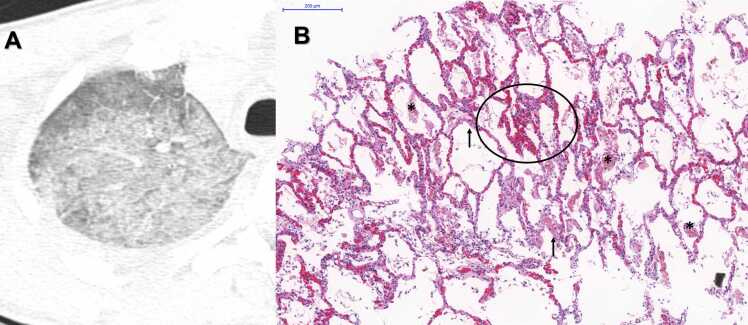
A) Postmortem Chest CT Image, Axial View, Right Upper Regions: There is a considerable amount of ground-glass opacities. B) Histopathological sample of the same regions: Photomicrograph of Acute Exudative DAD showing moderate septal inflammation, intense vascular congestion (ellipse), intra-alveolar fibrin deposition (asterisk) and formation of hyaline membranes (arrows) - HE Staining. Scale Bar - 200 µm - Objective 10x.

**Fig. 9:**
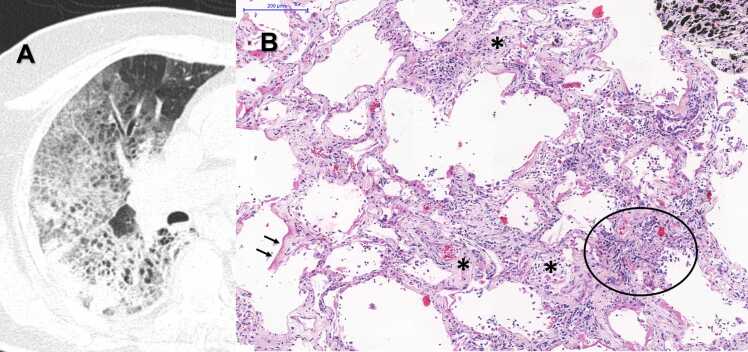
A) Postmortem Chest CT Image, Axial View, Right Upper Regions: There is a considerable amount of ‘crazy paving’ (thickened interlobular septa and intralobular lines superimposed on a background of ground-glass opacities). B) Histopathological sample of the same regions: Photomicrograph showing mixed DAD with some characteristics of acute DAD such as hyaline membranes (arrows) and abundant inflammatory cells (ellipse), as well as of fibroproliferative DAD with collagen deposition forming intra-alveolar plugs and septal thickening (asterisk) - HE Staining. Scale Bar - 200 µm - Objective 10x.

**Fig. 10:**
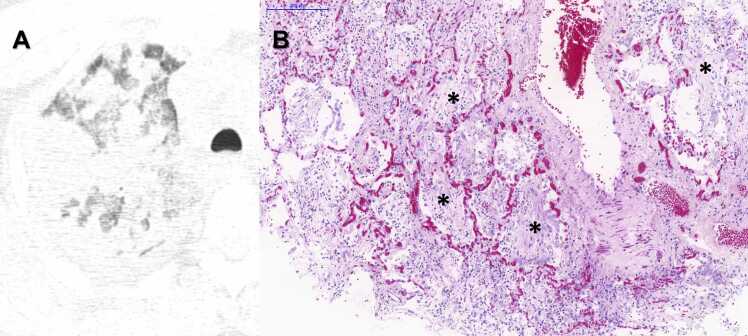
A) Postmortem Chest CT Image, Axial View, Right Upper Regions: There is a considerable amount of lung consolidations – small and large. B) Histopathological sample of the same regions: Photomicrograph of intense fibroproliferative DAD showing collagen deposition forming intra-alveolar plugs throughout the slide (asterisk) - HE Staining. Scale Bar - 200 µm - Objective 10x.

Some specific examples of postmortem chest CT lung images and the corresponding histopathological findings of our sample are presented in Fig. 7, Fig. 8, Fig. 9, Fig. 10. The other quantitative and qualitative fatal COVID-19 postmortem chest CT findings of the patients are shown in Table 3.

**Table 3:** Other quantitative and qualitative fatal COVID-19 postmortem chest CT findings.

Global Lung Involvement	
80 to 100 %	11/15 (73.33 %)
60 to 80 %	3/15 (20.00 %)
40 to 60 %	1/15 (6.67 %)
< 40 %	None
**Uni/Bilateral**	
Bilateral	15/15 (100 %)
Unilateral	None
**Affected Lobes**	
Only lower lobe(s)	None
Lower lobe(s) + at least one other lobe	15/15 (100 %)
No lower lobe involvement	None
**Predominance**	
Inferior	1/15 (6.67 %)
Posterior	3/15 (20.00 %)
No predominance	11/15 (73.33 %)
**Distribution**	
Peripheral	None
Central	None
Mixed	15/15 (100 %)
**Pulmonary Emphysema**	1/15 (6.67 %)
**Pulmonary Fibrosis**	None
**Mediastinal Lymph Node Enlargement**	8/15 (53.33 %)
**Pleural Effusion**	
Right	
Large	None
Moderate	2/15 (13.33 %)
Small	5/15 (33.33 %)
None	8/15 (53.33 %)
Left	
Large	None
Moderate	3/15 (20.00 %)
Small	5/15 (33.33 %)
None	7/15 (46.66 %)
**Pleural Thickening**	3/15 (20.00 %)
**Pneumothorax**	3/15 (20.00 %)
**Pericardial Effusion**	2/15 (13.33 %)
**Pneumomediastinum**	None
**Aortic Calcifications**	7/15 (46.66 %)
**Coronary Calcifications**	6/15 (40.00 %)
**Subcutaneous emphysema**	None

## 4 Discussion

One of the primary factors to be considered pertains to the utility of minimally invasive autopsy in the presented situation. The severity index score created relies on both the percentage of disease involvement and the progression/severity of the disease. As the CT severity score index advances, there is a corresponding progression in the histopathological correlated index.

The utility of chest CT in living patients with COVID-19 was very well established for diagnosis and progression of the disease [8], [14], [15]. Regarding postmortem chest CT in COVID-19, since the first case reports were published, attempts have been made to correlate tomographic findings with the progression/severity of the disease, as well as with what would be expected to be found in histopathological findings. Published data suggest that fatal COVID-19 cases exhibit extensive consolidations on CT scans and advanced DADs on histopathological slides, as endorsed by some authors [16], [17], [18], [19].

To date, most published studies have been case reports or series of case reports [10], [19], [20], [21]. This may be due to the difficulty of medical institutions having a dedicated CT scanner for postmortem studies. Additionally, the urgency to study and share information about COVID-19, especially during the early waves of the pandemic, may have limited the conduction of large casuistic studies, which usually requires more time. Despite the known limitations of postmortem chest CT [22], studies comparing COVID-19 pre and postmortem chest CT findings showed that comparison between them is possible [10], [11]. A meta-analysis regarding postmortem chest CT in fatal COVID-19 cases was performed and was published in 2022 to try to understand how and what importance that postmortem CT has [16], and it selected a few studies that compared postmortem CT versus histopathological findings (8 out of 20).

The decision to use the mean CT and histopathological severity score indexes for each patient and not for each region was a way to reduce the limitation that it was not always possible to determine the exact limits of each region in both analyses (CT and histopathological), mainly because tissue sample collections were sometimes made with placing the needle with an oblique entry angle, the adjacent region could have been sampled, the fact that the biopsies were sampled under ultrasound guidance and not with CT guidance, and there was a pairwise discordance between CT and histology. This method enabled an increase in the signal-to-noise ratio and demonstrated a positive correlation between the mean CT and Histopathological Severity Score Indexes (R = 0.66, p = 0.0078 – Fig. 5), showing that as the disease progressed with regard to pathology, whether in severity or extent, and that the disease also progressed in postmortem CT, despite the specific findings of the different phases of the disease not showing concordance between CT and histological slides. It is important to consider that a histological slide represents only a small sample of tissue, while the CT analysis includes larger regions of the lung. This “representation” difference imposes additional challenges for direct correlation between pathology and CT in a given region. An exact point-to-point correlation with CT-guided biopsies (which was not the case) could have improved these results, as suggested in other non-COVID correlation studies.

We also found a positive correlation between normal lung in CT and in the slides (ρ = 0.65; p = 0.0082) and a trend in the correlation between the proportion of normal lung tissue decreasing in histological analysis, as consolidation extended (ρ = 0.51; p = 0.055). This leads us to believe that the use of postmortem chest CT might be useful for screening cases without lung involvement. In other words, postmortem CT appears to be a good strategy for excluding disease, corroborating on what other authors had already suggested in initial case reports, as referred to previously in this text. The presence of large consolidations corroborates that the lungs are histologically affected (nonnormal). Future studies might evaluate the probable high negative predictive value of postmortem CT scans.

In addition, our data showed an intriguing relationship between the mean proportion of ground-glass opacities and the histological categories. We found a positive correlation between the mean proportion of ground-glass opacities and the proportion of normal lung tissue and a negative correlation between the proportion of ground-glass opacities and the percentage of disease in the exudative phase. One possible explanation for these findings is that in postmortem cases, the value of ground-glass opacities as a predictor of disease may be questioned once the images are obtained with exhaled lungs and there is more blood stasis, generating nonpathological ground-glass opacities. This is a limitation of the method, as stated previously. Furthermore, as is known in the chest CT of living individuals, when expiration series are performed to evaluate areas of air trapping, we notice that normal areas (without trapping) end up exhibiting high attenuation, simulating ground-glass opacities [12], [23]. Perhaps this is the greatest limitation of postmortem chest CTs. Since the lungs are most often in expiration, ground-glass opacities should be interpreted with caution. Some authors have already tried to differentiate postmortem ground-glass opacities attributed to COVID-19 from high attenuation, ground-glass mimicking opacities that would only be related to death itself and not COVID-19 infection [10]. They concluded that fatal COVID-19 ground-glass opacities correlate with bilateral, peripheral and multilobar distribution, as in life, despite the small number of cases. In our sample of 15 patients, most of our patients had large consolidations occupying considerable lung volumes, sometimes even encompassing the entire lobe, and most of our ground-glass/high attenuation opacities did not follow these typical viral disease patterns, probably explaining why we found a positive correlation between ground-glass opacities on CT and normal lungs tissue on the slides. A suggestion would be to suspect ground-glass opacities with the classic distribution of COVID-19, more peripheral, basal and multilobar [8], as pathological, and not those with atypical distribution, which would be more likely related to lung expiration. Another option for future studies regarding these ground-glass opacities confounding factors in postmortem studies could be the use of modern techniques such as CT radiomics analysis. Some authors used it as a supplementary tool for improving specificity for COVID-19 in a living population confounded by ground glass opacity changes from other etiologies [24].

One way to diminish those postmortem chest CT expiration-related issues would be to have patients undergo tracheal intubation or crico-thyrioidotomy [25], insufflate the lungs, and then perform the scan. Two possible issues are known: the difficulty of orotracheal intubation due to head and neck death-related rigor of the soft tissues, as well as possible gas leakage in different body compartments [26]. As our study was conducted in the first wave of the pandemic, very little was known about the virus; thus, it was decided to scan patients without intubation to minimize team contagion risks.

The major limitation of minimally invasive autopsy is that other contributors to death (such as acute myocardial infarct or pulmonary thromboembolism) could not be evaluated as they would been evaluated during conventional autopsy. Despite this fact, the method enabled us to improve the understanding of radiological findings and disease progression.

As future lines of studies, larger projects integrating diverse imaging modalities (such as ultrasound and magnetic resonance) also across other parts of the human body, hold promise in enhancing the applicability of minimally autopsy methods, not only in fatal COVID-19. Additionally, the incorporation of artificial intelligence and machine learning in the analysis of postmortem CT images, as it is already being used in living patients [24], [27], has the potential to significantly enhance the precision and interpretation of imaging, especially if a large histopathological dataset is input.

## 5 Conclusions

The use of a minimally invasive autopsy method was useful in accessing the lungs of fatal COVID-19 patients by imaging and histopathology, especially in a context of restricted or suspended conventional autopsies. There is a correlation between the progression and severity of the disease when comparing postmortem CT and histopathology findings. Furthermore, encountering normal lungs in postmortem chest CT might be a good indication that those lungs are also normal in histopathology, and the interpretation of ground-glass opacities should be done with caution.

## Funding

This work was supported by the 10.13039/100000865Bill and Melinda Gates Foundation [INV‐002396]; 10.13039/501100003593Conselho Nacional de Desenvolvimento Científico e Tecnológico [401825/2020-5, 304987/2017-4 to M.D. and 304277/2019-3 to T.M.]; 10.13039/501100001807Fundação de Amparo à Pesquisa do Estado de São Paulo [2013/17159‐2]; and Hospital das Clinicas da Faculdade de Medicina da Universidade de São Paulo - HC Convida [HC-01.18/2020, HC-01.29/2020, and HC-02.18/2020].

## Ethical statement

This study was approved by our National Research Ethical Committee (CONEP CAAE 30364720.0.0000.0068). Informed consents were obtained from the next of kin of all deceased patients.

## CRediT authorship contribution statement

**Saldiva Paulo Hilário Nascimento:** Writing – review & editing, Validation, Supervision, Methodology, Investigation, Formal analysis, Data curation, Conceptualization. **Mauad Thais:** Writing – review & editing, Validation, Supervision, Project administration, Methodology, Funding acquisition, Formal analysis, Conceptualization. **Dolhnikoff Marisa:** Writing – review & editing, Validation, Supervision, Project administration, Methodology, Funding acquisition, Formal analysis, Conceptualization. **Martin Maria da Graça Morais:** Writing – review & editing. **Savoia Paulo:** Writing – review & editing, Writing – original draft, Visualization, Validation, Project administration, Methodology, Investigation, Funding acquisition, Formal analysis, Data curation, Conceptualization. **Cardoso Ellison Fernando:** Writing – review & editing, Writing – original draft, Validation, Supervision, Resources, Project administration, Methodology, Investigation, Funding acquisition, Formal analysis, Data curation, Conceptualization. **da Silva Luiz Fernando Ferraz:** Writing – review & editing, Validation, Supervision, Resources, Project administration, Methodology, Investigation, Funding acquisition, Formal analysis, Data curation, Conceptualization. **Leite Claudia da Costa:** Writing – review & editing, Validation, Supervision, Resources. **Duarte-Neto Amaro Nunes:** Writing – review & editing, Methodology, Formal analysis. **Monteiro Renata Aparecida Almeida:** Writing – review & editing, Methodology. **Sawamura Marcio Valente Yamada:** Writing – review & editing, Validation, Supervision, Formal analysis.

## Declaration of Competing Interest

The authors declare the following financial interests/personal relationships which may be considered as potential competing interests: Marisa Dolhnikoff reports financial support was provided by Bill and Melinda Gates Foundation. Marisa Dolhnikoff reports financial support was provided by Conselho Nacional de Desenvolvimento Científico e Tecnológico. Thais Mauad reports financial support was provided by Conselho Nacional de Desenvolvimento Científico e Tecnológico. Marisa Dolhnikoff reports financial support was provided by Fundação de Amparo a Pesquisa do Estado de São Paulo (FAPESP). Ellison Fernando Cardoso reports financial support was provided by University of Sao Paulo Hospital of Clinics. Luiz Fernando Ferraz da Silva reports financial support was provided by University of Sao Paulo Hospital of Clinics.
